# Genetic Selection of Peptide Aptamers That Interact and Inhibit Both Small Protein B and Alternative Ribosome-Rescue Factor A of *Aeromonas veronii* C4

**DOI:** 10.3389/fmicb.2016.01228

**Published:** 2016-08-18

**Authors:** Peng Liu, Yong Chen, Dan Wang, Yanqiong Tang, Hongqian Tang, Haichao Song, Qun Sun, Yueling Zhang, Zhu Liu

**Affiliations:** ^1^Department of Biology, College of Sciences, Shantou UniversityShantou, China; ^2^Hainan Key Laboratory for Sustainable Utilization of Tropical Bioresources, College of Agriculture, Hainan UniversityHaikou, China; ^3^Department of Biotechnology, College of Life Sciences, Sichuan UniversityChengdu, China

**Keywords:** peptide aptamers, SmpB, ArfA, interaction, genetic selection

## Abstract

*Aeromonas veronii* is a pathogenic gram-negative bacterium, which infects a variety of animals and results in mass mortality. The stalled-ribosome rescues are reported to ensure viability and virulence under stress conditions, of which primarily include trans-translation and alternative ribosome-rescue factor A (ArfA) in *A. veronii*. For identification of specific peptides that interact and inhibit the stalled-ribosome rescues, peptide aptamer library (pTRG-SN-peptides) was constructed using pTRG as vector and *Staphylococcus aureus* nuclease (SN) as scaffold protein, in which 16 random amino acids were introduced to form an exposed surface loop. In the meantime both Small Protein B (SmpB) which acts as one of the key components in trans-translation, and ArfA were inserted to pBT to constitute pBT-SmpB and pBT-ArfA, respectively. The peptide aptamer PA-2 was selected from pTRG-SN-peptides by bacterial two-hybrid system (B2H) employing pBT-SmpB or pBT-ArfA as baits. The conserved sites G_133_K_134_ and D_138_K_139_R_140_ of C-terminal SmpB were identified by interacting with N-terminal SN, and concurrently the residue K_62_ of ArfA was recognized by interacting with the surface loop of the specific peptide aptamer PA-2. The expression plasmids pN-SN or pN-PA-2, which combined the duplication origin of pRE112 with the neokanamycin promoter expressing SN or PA-2, were created and transformed into *A. veronii* C4, separately. The engineered *A. veronii* C4 which endowing SN or PA-2 expression impaired growth capabilities under stress conditions including temperatures, sucrose, glucose, potassium chloride (KCl) and antibiotics, and the stress-related genes *rpoS* and *nhaP* were down-regulated significantly by Quantitative Real-time PCR (qRT-PCR) when treating in 2.0% KCl. Thus, the engineered *A. veronii* C4 conferring PA-2 expression might be potentially attenuated vaccine, and also the peptide aptamer PA-2 could develop as anti-microbial drugs targeted to the ribosome rescued factors in *A. veronii*.

## Introduction

*Aeromonas veronii* is a type of important pathogenic microbes to human and aquatic animals (Graf, 1999), which is classified as gram-negative bacterium and distributed in freshwater and sewage (Sugita et al., 1995). *A. veronii* produces various virulence factors, such as cytotoxic enterotoxin (Act), aerolysin (AerA), lipase (Lip), flagellin (Fla), and out membrane protein (OmpA) (Namba et al., 2008; Nawaz et al., 2010), thereby inducing wound infections, dysentery and septicemia in immunocompromised individuals (Graf, 1999; Cui et al., 2007; Gröbner et al., 2007), and likewise causing bacterial septicemia in fish and inflicting grievous economic harm in the aquaculture industry (Li et al., 2011). In aquaculture, the antibiotics are mainly applied to prevent and cure *A. veronii* pathogen. However, widespread consumptions of antibiotics in fish farming result in more and more multidrug resistant strains of *A. veronii* (Varela et al., 2016), which resist to tetracycline, ampicillin, and streptomycin (Castro-Escarpulli et al., 2003; Nawaz et al., 2006). The emergence of multidrug resistant strains of *A. veronii* in fish farming should not be ignored, as they not only seriously impact on the economic benefit of aquaculture industry, but also jeopardize the health and life of human beings (Castro-Escarpulli et al., 2003; Yücel and Balci, 2010). Therefore, the development of new antibiotics and protein drugs is becoming an exigent task.

Recent studies reveal that the stalled-ribosome rescues play crucial roles for pathogenic survival and virulence, especially under the stress conditions. Among these rescue systems, trans-translation which is mediated by transfer-messenger RNA (tmRNA) and the associated Small Protein B (SmpB) is predominant and essential for translation quality control to the vast majority of bacteria (Keiler, 2015). However, the deficiencies of tmRNA and SmpB do not hamper the growth in *E. coli*, thereby suggesting a far more complicated ribosome rescue events (Chadani et al., 2010). Subsequently the alternative ribosome rescue factors alternative ribosome-rescue factor A (ArfA) and YaeJ are discovered to ensure cell viability and virulence under stress conditions (Giudice and Gillet, 2013). As a matter of fact, many pathogenic species, of which include *Mycobacterium tuberculosis, Yersinia pseudotuberculosis, Salmonella Typhimurium, Shigella flexneri* (Okan et al., 2006; Ansong et al., 2009; Shi et al., 2011; Ramadoss et al., 2013), have multiple rescue systems when confronting to stalled translation events (Himeno et al., 2015). Hence stalled-ribosome rescue factors are predominant for bacterial growth and survival because of their ubiquity and essentiality.

The discovery of novel antibacterial agents usually starts to exploit attractive pathways, and then screens new specific agents that inhibit the targets (Lange et al., 2007). For inhibition of pathogenic bacteria, the pathways of stalled-ribosome rescues are considered as candidates of interest with the following reasons. First, the rescue factors referring to tmRNA, SmpB, ArfA, and YaeJ are not appeared in animal cells, resulting in low side effects to animals when employing the selected anti-microbial drugs targeted to the systems (Vioque and de la Cruz, 2003). Secondly, trans-translation system mediated by tmRNA and SmpB is important for persister survival and tolerance to a variety of antibiotics and stresses (Li et al., 2013), and tuberculosis drug pyrazinamide inhibits the trans-translation leading to specifically kill the persisters in *Mycobacterium tuberculosis* (Shi et al., 2011). In clinical researches, the pathogenic persisters which keep at a dormant state are recalcitrantly produced when treating with antibacterial agents (Chowdhury et al., 2016), and will recover to chronic and long-term infections after drugs elimination (Wang et al., 2015), which posing a serious challenge to the antimicrobial treatments (Keren et al., 2004). Thus, stalled-ribosome rescue pathways involved in SmpB and ArfA are attractive targets for anti-microbial agents with the inhibition of *A. veronii*.

For this purpose, the peptide aptamers were selected to interact with SmpB and ArfA. The peptide aptamers are combinatorial proteins consisting of a stable scaffold protein and random amino acids, which typically embed as an exserted surface loop (Reverdatto et al., 2015). When the peptide aptamer binds to the target with high affinity and specificity, it inactivates the target protein at the intracellular level (Hoppe-Seyler et al., 2004). Now the peptide aptamers have emerged as a useful molecular tool that is applied in aspects of molecular medicine (Colombo et al., 2015). In this study, the peptide aptamer library was designed using *Staphylococcus aureus* nuclease (SN) as scaffold protein on which inserting 16 random amino acids to shape as a loop. In this study, the peptide aptamer PA-2 was isolated and demonstrated to interact specifically with both SmpB and ArfA by bacterial two-hybrid assay (B2H), resulting to dysfunction of both SmpB and ArfA, and subsequently intensifying the sensitivities under the conditions of temperatures, glucose, sucrose, potassium chloride, and antibiotics. Collectively, not only the peptide aptamer PA-2 could be as anti-microbial drugs targeted to the ribosome rescued factors, but also the transformant conferring PA-2 was potentially attenuated vaccine for the prevention of *A. veronii*.

## Materials and methods

### Constructions of the plasmids

Plasmids used in this study were showed in Table 1, and all used primers were listed in Supplementary Table 1, and the sequences of SN and SmpB and ArfA were provided in Supplementary Figures 1–3, respectively. The PCR amplification was performed as follows: 94°C for 3 min, followed by 94°C for 30 s, 55–60°C for 30 s and 72°C for 1 kb/min in a total of 30 cycles. For construction of pBT-ArfA, a 240-bp DNA fragment containing the encoded region of *arfA* was amplified from the genomic DNA of *A. veronii* C4 with primers F1/R1, carrying *Eco* RI/*Bgl* II at the 5′-end respectively. And then purified PCR product was digested by *Eco* RI/*Bgl* II, and inserted into “bait” vector pBT to yield pBT-ArfA. For constructions of pTRG-SN and its truncations, the fragments were amplified by PCR using the plasmid pTCN-22 as template and the primers F2/R2, F3/R3, F4/R3, and F4/R4, respectively. Subsequently the purified PCR product was digested by *Bam* HI/*Spe* I, and inserted into pTRG (Stratagene, California, USA) to generate pTRG-SN, pTRG-SNΔC45, pTRG-SNΔN57C45, and pTRG-SNΔN57 (Liu et al., 2015). For constructions of the mutants derived from pBT-ArfA, site-directed mutagenesis was implemented according to the protocol supported by QuickChange Kit (Qiagen, Shenzhen, China). In brief, for constructing pBT (ArfA-E_28_I_29_K_30_), in which the residues E28I_29_K_30_ were substituted to alanines, PCR was performed using pBT-ArfA as template and F12/R12 as the primers to which the specific mutations were designed and introduced. In the reaction, 1 μl of DNA template (400 pg/μl), 1 μl primer either F12 or R12 (10 mM), 25 μl PrimeSTAR mix (Takara, Dalian, China) were included in 50 μl volume for running 15 cycles, followed by a combination of both PCR products for an additional 15 cycles. Eventually the purified PCR was digested with *Dpn* I and transformed into *E. coli*. The mutated plasmids were extracted from positive colonies and verified by DNA sequencing (SangonBiotech, Shanghai, China). Similarly, a series of pBT-ArfA mutants including pBT (ArfA-D_31_N_32_), pBT (ArfA-A_36_L_37_), pBT (ArfA-L_42_F_43_), pBT (ArfA-K_52_G_53_K_54_), pBT (ArfA-G_55_S_56_Y_57_), pBT (ArfA-R_59_K_60_), and pBT (ArfA-K_62_) were constructed in which the residues were substituted to alanine except that K_62_ was substituted to proline (Smith, 2007; Liu et al., 2015). For construction of pN-NeoR, a 933-bp DNA fragment containing 138-bp *neoR* promoter and 795-bp encoding region of *neoR* fragment was amplified from plasmid pk18mobsacB using primers F5/R5 carrying *Xma* I/*Xba* I sites at the 5′-end, respectively. The purified PCR product was digested, and inserted into pRE112 to yield pN-NeoR. For construction of pN-SN, 138-bp *neoR* promoter and 552-bp encoding region of *Staphylococcal* nuclease gene were amplified from pN-NeoR and pTRG-SN using primers F6/R6 and F7/R7, separately, and then both purified fragments were combined in an overlap extension PCR reaction using F6/R7 (Heckman and Pease, 2007), and purified and digested by *Xma* I/*Xba* I, and inserted into pRE112 to yield pN-SN (Ho et al., 1989). The plasmid pN-PA-2 was constructed using F6/R7 in similar manner.

**Table 1:** **Plasmids used in this study**.

**Plasmids name**	**Properties**	**Source or References**
pBT-LGF2	Positive control, 3.3 kb plasmid, p15A ori, lac UV-5 promoter, Cam^R^.	Stratagene
pTRG-Gal11^p^	Positive control, 4.6 kb plasmid, ColE1 ori, *lpp/lac*-UV5 promoter, Tet^R^.	Stratagene
pBT	Bait plasmid, 3.2 kb, p15A ori, lac UV-5 promoter, Cam^R^.	Stratagene
pTRG	Prey plasmid, 4.4 kb, ColE1 ori, *lpp/lac*-UV5 promoter, Tet^R^.	Stratagene
pBT-ArfA	pBT derivative, expresses ArfA with λcI, CamR.	This study
pBT (ArfA-E28IK)	pBT-ArfA derivative, mutates E28IK to AAA.	This study
pBT (ArfA-D31N)	pBT-ArfA derivative, mutates D31N to AA.	This study
pBT (ArfA-A36L)	pBT-ArfA derivative, mutates A36L to AA.	This study
pBT (ArfA-L42F)	pBT-ArfA derivative, mutates L42F to AA.	This study
pBT (ArfA-K52GK)	pBT-ArfA derivative, mutates K52GK to AAA.	This study
pBT (ArfA-G55SY)	pBT-ArfA derivative, mutates G55SY to AAA.	This study
pBT (ArfA-R59K)	pBT-ArfA derivative, mutates R59K to AA.	This study
pBT (ArfA-K62)	pBT-ArfA derivative, mutates K62 to P.	This study
pBT-SmpB	pBT derivative, expresses *smpB* with λ cI.	Liu et al., 2015
pBT-SmpBΔN34	pBT-SmpB derivative, deletes 34-residue at N-terminal SmpB.	Liu et al., 2015
pBT-SmpB ΔN34C30	pBT-SmpB derivative, deletes 34-residue at N-terminal and 30-residue at C-terminal SmpB.	Liu et al., 2015
pBT-SmpBΔC30	pBT-SmpB derivative, deletes 30-residue at C-terminal SmpB.	Liu et al., 2015
pBT (SmpB-G11S)	pBT-SmpB derivative, mutates G11S to AA.	Liu et al., 2015
pBT (SmpB-T14I)	pBT-SmpB derivative, mutates T14I to AA.	Liu et al., 2015
pBT (SmpB-F26I)	pBT-SmpB derivative, mutates F26I to AA.	Liu et al., 2015
pBT (SmpB-E32AG)	pBT-SmpB derivative, mutates E32AG to AAA.	Liu et al., 2015
pBT (SmpB-G133K)	pBT-SmpB derivative, mutates G133K to AA.	Liu et al., 2015
pBT (SmpB-D138KR)	pBT-SmpB derivative, mutates D138KR to AAA.	Liu et al., 2015
pBT (SmpB-K152)	pBT-SmpB derivative, mutates K152 to P.	Liu et al., 2015
pTCN-22	7.1-kb plasmid, expresses SN with six histidine residues, ColE1 ori, Amp^R^.	Norman et al., 1999
pTRG-SN	pTRG derivative, expresses SN with RNAP, Tet^R^.	This study
pTRG-SNΔN57	pTRG derivative, deletes 57-residues at N-terminus of SN.	This study
pTRG-SN ΔN57ΔC45	pTRG derivative, deletes 57-residues at N-terminus, and 45-residues at the C-terminus of SN.	This study
pTRG-SNΔC45	pTRG derivative, deletes 45-residues at C-terminus of SN.	This study
pTRG-SN-peptides	pTRG derivative, expresses random peptide with RNAP, Tet^R^.	This study
pTRG-PA-2	pTRG derivative, expresses SN-PA-2 with RNAP, Tet^R^.	This study
pRE112	5.8-kb suicide plasmid, with oriT RP4, Cam^R^.	Edwards et al., 1998
pk18mobsacB	5.7-kb suicide plasmid, with pBR322 ori and neokanamycin promoter, NeoR, Kan^R^.	Schäfer et al., 1994
pN-NeoR	pRE112 derivative, inserted neokanamycin promoter, and NeoR of pk18mobsacB	This study
pN-SN	pRE112 derivative, expresses SN under control of pk18mobsacB neokanamycin promoter.	This study
pN-PA-2	pRE112 derivative, expresses SN-PA-2 under control of pk18mobsacB neokanamycin promoter.	This study

### Strains and growth conditions

Bacterial strains used in this study were listed in Table 2. *E. coli* XL1-Blue MRF' was used to propagate pBT and pTRG derivatives. *E. coli* XL1-Blue MR which owned reporter genes *his*3 and *aadA* was used for bacterial two-hybrid (B2H) analysis (Joung et al., 2000). *E. coli* strains were grown routinely at 37°C in Luria-Bertani (LB) medium. All reagents were purchased from Stratagene and B2H assay was performed according to the instruction manual of BacterioMatch II Two-Hybrid System Vector Kit (Stratagene, California, USA).

**Table 2:** **Bacterial strains used in this study**.

**Strains name**	**Genotype, and/or relevant feature(s)**	**Source or References**
*Aeromonas veronii* C4	Wild type, ampicillin resistance, virulent to *Ctenopharyngodon idella*.	Liu et al., 2015
*Aeromonas veronii* C4 (pRE112)	The engineered *A. veronii* C4 carries pRE112 empty vector.	This study
*Aeromonas veronii* C4 (pN-SN)	The engineered *A. veronii* C4 expresses SN by pN-SN recombinant plasmid.	This study
*Aeromonas veronii* C4 (pN-PA-2)	The engineered *A. veronii* C4 expresses PA-2 by pN-PA-2 recombinant plasmid.	This study
*E. coli* WM3064	*thrB 1004 pro thi rpsL hsdS lacZ*ΔM15 RP4-1360 Δ(*araBAD)567 ΔdapA1341::[erm pir]*.	Edwards et al., 1998
*E. coli* XL1-Blue MRF'	*Δ(mcrA)183 Δ(mcrCB-hsdSMR-mrr)173 endA1 supE44 thi-1 recA1 gyrA96 relA1 lac* [F'*proAB lacIqZΔM15 Tn5* (Kanr)].	Stratagene
*E. coli* XL1-Blue MR	*Δ(mcrA)183 Δ(mcrCB-hsdSMR-mrr)173 endA1 hisB supE44 thi-1 recA1 gyrA96 relA1 lac* [F'*lacIq HIS3 aadA* Kanr].	Stratagene
*Aeromonas veronii* C4 Δ*smpB*	The *smpB* gene is deleted from wild type, Ampicillin resistance.	Liu et al., 2015

The strain *A. veronii* C4 was isolated from *Ctenopharyngodon idella* (Liu et al., 2015), and grown at 30°C on LB agar plate with 50 μg/ml ampicillin. For the constructions of engineered *A. veronii* C4, the plasmids pRE112 and its derivatives pN-SN, pN-PA-2 were transformed into donor *E. coli* WM3064, and the positive clones were selected on LB agar supplemented with 0.3 mM diaminopimelic acid (DAP) and 25 μg/ml chloramphenicol at 37°C respectively (Edwards et al., 1998), followed by conjugation with *A. veronii* C4 (Saltikov and Newman, 2003). Successively the mating mixtures were selected on LB agar supplemented with 50 μg/ml ampicillin and 25 μg/ml chloramphenicol, and the engineered *A. veronii* C4 were picked and verified by PCR using primers F1/R1 and F8/R8. All the engineered *A. veronii* C4 were incubated at 30°C in LB with 50 μg/ml ampicillin and 25 μg/ml chloramphenicol.

### Construction of peptide aptamer library

The nuclease (SN) of *Staphylococcus aureus* was chosen as scaffold protein (Mascini et al., 2012). For randomly appending an exposed surface loop on SN to constitute peptide aptamer library (pTRG-SN-peptides), the single-stranded nucleotides were designed and synthesized as follows, 5′-CCGGAATTCGGT GGT(NNS)_16_GGTG GTAGATCTAAGTAC-3′, where N indicated a mixture of Adenine, Thymine, Cytosine and Guanine, and S indicated a mixture of Cytosine and Guanine (de Chassey et al., 2007). The primer extension was performed to produce double-stranded oligonucleotides by employing single-stranded oligonucleotides as template and F9/R9 as primers (Worthington et al., 2001). The optimal PCR condition was executed at 95°C for 90 s, followed by 95°C for 30 s, 46°C for 1 min and 72°C for 2 min in a total of 8 cycles, and a final extension at 72°C for 7 min. The double-stranded PCR product was cut with *Eco* RI/*Bgl* II, and inserted into pTRG-SN which digested with the same enzymes, yielding to pTRG-SN-peptides. After 1 μl of the ultimate constructs were transformed into 40 μl of DH5α competent cells by electroporation, the recovery cells were incubate at 37°C for 12 h and applied to calculate the library titer by counting the number of colony forming units (CFU).

### Selection of peptide aptamers

B2H was performed to select peptide aptamers interacting with both SmpB and ArfA from pTRG-SN-peptides. The genes *smpB* or *arfA* were cloned into the pBT bait to generate pBT-SmpB or pBT-ArfA, separately (Table 1). Both the baits pBT-SmpB or pBT-ArfA and the prey pTRG-SN-peptides were co-transformed into *E. coli* XL1-Blue MR. The transformants were cultivated at 37°C for 24 h in selective screen mediums containing 5 mM 3-amino-1, 2, 4-triazole (3-AT), 25 μg/ml chloramphenicol and 12.5 μg/ml tetracycline, and then continued to culture in dark location at room temperature for another 16 h (Zhou et al., 2015). The positive clones were picked and validated by PCR with the primers F10/R10 and F11/R11. For eliminating pBT-SmpB or pBT-ArfA to acquire pure peptide aptamers, the positive colony was cultured in LB supplemented with 12.5 μg/ml tetracycline at 37°C, 150 rpm for 16 h, and then plated onto LB agar containing only 12.5 μg/ml tetracycline for additional 16 h. The resulting colony was streaked onto LB agar containing 12.5 μg/ml tetracycline at 37°C for 12 h, followed by interspersing onto plates in which either containing 12.5 μg/ml tetracycline or 25 μg/ml chloramphenicol independently. Simultaneously the co-transformant of pBT-LGF2 and pTRG-Gal11^p^ was used as positive control, while the colony endowing with pBT or pTRG was employed as negative control (Dove and Hochschild, 2004).

### Analysis of functional regions and key interaction sites

To identify the functional regions and key interaction sites between SmpB and SN, pBT-SmpB mutants and pTRG-SN truncations were co-transformed into *E. coli* XL1-Blue MR, and grown in LB agar with 25 μg/ml chloramphenicol and 12.5 μg/ml tetracycline at 37°C for 24 h. The positive were inoculated in LB with appropriate antibiotics at 37°C, 150 rpm overnight, and sub-cultured with an initial OD_600_ of 0.02–0.5, and successively 10 μl of each culture were dotted on selective and non-selective medium using serial dilutions, and incubated at 37°C for 24 h, and transferred in dark location at 25°C for additional 16 h. Equally the functional regions and key interaction sites between ArfA and PA-2 were analyzed and identified (Martínez et al., 2014; Zhou et al., 2015).

### Protein structure modeling and molecular docking

The iterative threading assembly refinement (I-TASSER) server was applied to predict three-dimensional structure (Roy et al., 2010). I-TASSER modeling begins with the template structure sorted by LOMETS (Local Meta-Threading-Server) from PDB database (Zhang, 2008; Yang et al., 2015). The peptide aptamer sequences containing scaffold protein SN and exposed surface loop were submitted to I-TASSER server by comparing with the template *Staphylococcus aureus* SN (PDB code: 1jok) to determine 3D structure. Likewise, the structural model of either SmpB or ArfA was generated by submitting the amino acid sequence to the I-TASSER in comparison with SmpB (PDB code: 1k8h) or ArfA (PDB code: 2tmaA), accordingly. The output of I-TASSER was analyzed using Pymol version 1.4.1 (Pawlowski et al., 2014). HADDOCK (High Ambiguity Driven protein-protein DOCKing) was executed to dock SmpB and SN, ArfA and PA-2 separately (de Vries et al., 2010; van Zundert et al., 2016).

### Growth measurement in engineered *A. veronii* C4

The plasmid pRE112 and its derivatives were transformed into *E. coli* WM3064 and then transferred into *A. veronii* C4 by conjugation (Wu et al., 2015). The growth curves of engineered *A. veronii* C4 were measured by taking 1 ml of culture to perform OD_600_ readings at regular intervals. The culture conditions were altered as follows: different temperatures (25, 30, 42°C), glucose concentrations (5, 10, 15%), sucrose (5, 10, 15%), potassium chloride (KCl) concentrations (0.5, 2, 5, 12.5%), tetracycline concentrations (0.1, 0.2, 0.3, 0.4, 0.5 μg/ml), erythromycin concentrations (5, 10, 15, 50 μg/ml) and kanamycin concentrations (5, 10, 15, 20 μg/ml).

### Quantitative real-time PCR (qRT-PCR)

The wild type and engineered *A. veronii* C4 strains were cultured until to stationary phase at 30°C, 150 rpm in 5 ml LB complemented with 2.0% KCl. The amounts of RNA were isolated using the RNAiso Plus reagent according to the manufacturer's instructions (Takara, Dalian, China). Residual genomic DNA was eliminated by treating with 4 μl of DNase I (RNase-free; 5 U/μl). Eventually the amount of RNA were precipitated by 75% ethanol and re-suspended in 50 μl of RNase free water (Gao et al., 2016), and reverse-transcribed into first strands of cDNA using PrimeScript™ 1^ST^ Strand cDNA Synthesis Kit. The qRT-PCR tests were performed in triplicate with the SYBR® Premix DimerEraser™ by using LightCycler® 480 Real-Time PCR System (Roche Applied Science, USA). The qRT-PCR primer sequences were shown in Supplementary Table 2. The qRT-PCR mixture composed of 100 ng cDNA template, 2 μl of each 10 μM forward or reverse primers and 10 μl of 2 × SYBR® Premix DimerEraser (Takara, Dalian, China) in a final volume of 20 μl. The PCR conditions were as follows: 95°C for 30 s, followed by 95°C for 5 s, 55°C for 30 s and 72°C for 30 s in a total of 40 cycles. The threshold cycle (Ct) values for stress sigma factor (*rpoS*) and NhaP-type Na+/H+ antiporter (*nhaP*) were normalized utilizing 16S rRNA as internal standard. The relative expression level was calculated using the 2^−ΔΔCt^ method, where Δ*ΔCt* = (Ct target − Ct 16S rRNA)_Treatment_ − (Ct target − Ct 16S rRNA) _Control_ (Huang et al., 2015). Likewise, the transcription levels of *rpoS* and *nhaP* were compared in wild type and *smpB* knock-out strain (Liu et al., 2015).

### Statistical analysis

The statistical data were presented as mean ± standard deviation in triplicate. Multiple comparisons were analyzed by one-way analysis of variance (ANOVA) with Statistical Package for the Social Sciences (SPSS) version 19.0 software (IBM, Armonk, NY, USA) and GraphPad Prism 5 software (San Diego, CA, USA). *P*-values less than 0.05 and 0.01 represented significant and extremely significant differences, respectively (Leskela et al., 2015).

## Results

### Construction of the random peptide aptamer library

**Figure 1:**
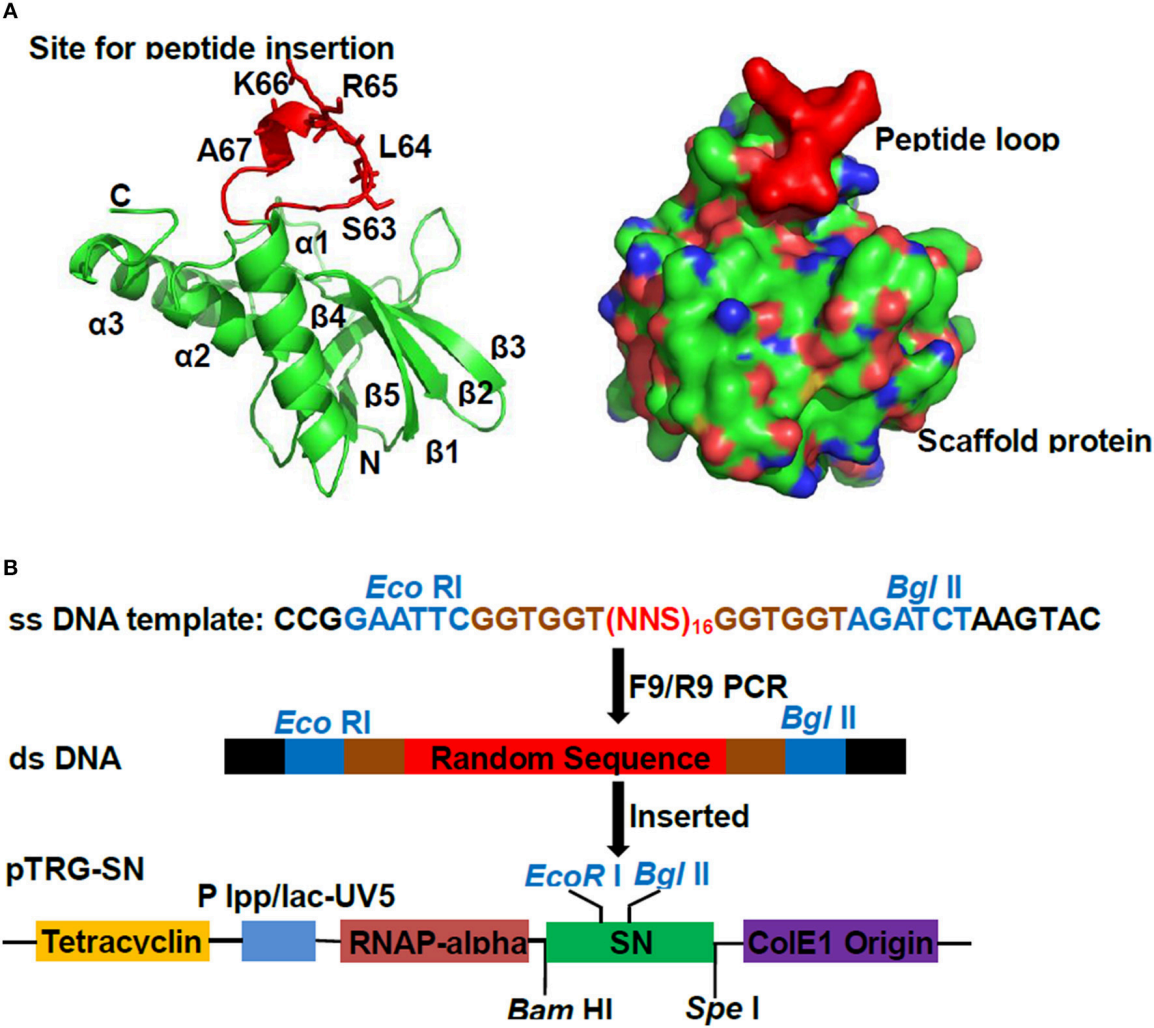
**Construction of peptide aptamer library**. **(A)** Ribbon diagrams of *Staphylococcus aureus* nuclease (SN) protein. The scaffold protein SN consisted of five β-strands and three helices. The sequences S_63_L_64_R_65_K_66_A_67_ which displaying the exserted loop was shown as scarlet stick representation. **(B)** The scheme for cloning random sequences to pTRG-SN vector. The double-stranded DNA were produced by employing single-stranded oligonucleotides containing randomized (NNS)_16_ nucleotide triplets as templates, followed by the digestions with *Eco* RI/*Bgl* II, and ligated into plasmid pTRG-SN which pre-cut with same enzymes.

To create aptamer library, a catalytically inactive version of SN was chosen as scaffold protein, wherein the residues S_63_L_64_R_65_K_66_A_67_ of SN were replaced by 16 random amino acids exhibiting surface loop (Figure 1A). The double-stranded oligonucleotides were synthesized, in which contain 48 random nucleotides flanked by *Eco* RI /*Bgl* II sites (Figure 1B). For getting the good yields and qualities of double strands, the cycles and temperatures of PCR reactions were optimized, and the resulting PCR products were verified on agarose gels with stained ethidium bromide (EB) (Supplementary Figure 4). The duplex products were purified and digested with *Eco* RI/*Bgl* II, and inserted into pTRG-SN to create the pTRG-SN-peptides (Figure 1B). After pTRG-SN-peptides were transformed into competent *E. coli* XL1-Blue MRF', the library titer was determined as 1.5–2.0 × 10^7^ CFU/ml, which was sufficient for subsequent library screen.

### Identification of functional regions and key interaction sites between SN and SmpB

**Figure 2:**
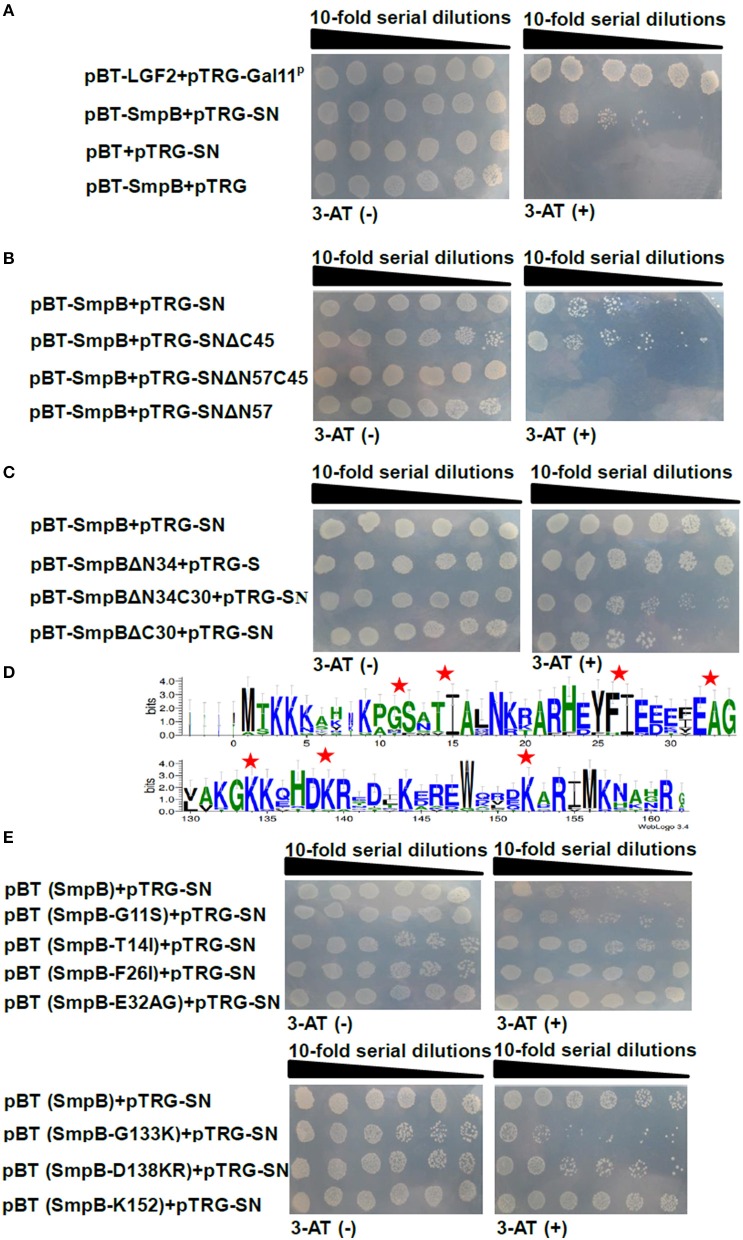
**Interaction between SmpB and SN by B2H**. The co-transformants were spotted onto non-selective and 5 mM 3-AT selective medium by series dilution method, on which 10 μl of each dilution was spotted. **(A)** Evaluation of SN interacting with SmpB. **(B)** Evaluation of SN truncations interacting with SmpB. **(C)** Identification of the binding regions of SmpB interacting with SN. **(D)** Alignments of N- and C- terminuses of 15 SmpB sequences (Supplementary Table 3) were displayed by WebLogo (http://weblogo.berkeley.edu/logo.cgi). The mutation sites are marked by red pentagrams. **(E)** Identification of the key sites of SmpB interacting with SN. The conserved amino acids of SmpB were mutated and applied for testing the interaction with SN.

B2H was employed to identify SN to interact with SmpB. The co-transformants endowing with pBT-SmpB and pTRG-SN appeared the abundance of colonies on 3-AT medium, whereas the colonies conferring pBT and pTRG-SN, pBT-SmpB and pTRG did not survive, revealing that only SN interacted with SmpB (Figure 2A). To determine the functional regions of SN interacting with SmpB, a series of SN truncations thereof were constructed, including the pTRG-SNΔC45, pTRG-SNΔN57, and pTRG-SNΔN57C45. As shown in Figure 2B, the co-transformants of pTRG-SNΔC45 and pBT-SmpB grew well on 5 mM 3-AT medium, while the co-transformants of pTRG-SNΔN57 or pTRG-SNΔN57C45 combining with pBT-SmpB did not, indicating that SmpB interacted with the headmost 57 residues of N-terminal SN. To analyze the critical regions of SmpB interacting with SN, a series of SmpB truncations were constructed, including pBT-SmpBΔN34, pBT-SmpBΔN34C30, and pBT-SmpBΔC30. The results showed that SN mainly interacted with C-terminus of SmpB (Figure 2C). For further identification of the key sites of SmpB interacting with SN, the conservative sites of SmpB were aligned from different pathogenic bacteria (Supplementary Table 3) using WebLogo (Figure 2D), and then site-directed mutagenesis were performed to substitute alanine for the conserved residues G_11_S_12_, T_14_I_15_, F_26_I_27_, E_32_A_33_G_34_, G_133_K_134_, D_138_K_139_R_140_, except for mutating K_152_ to proline (Smith, 2007), resulting in a series of pBT-SmpB mutants (Table 1). As shown in Figure 2E, the co-transformants of pBT-SmpB (G_11_S_12_) and pTRG-SN, pBT-SmpB (G_133_K_134_) and pTRG-SN, or pBT-SmpB (D_138_K_139_R_140_) and pTRG-SN had growth defects on 5 mM 3-AT medium, indicating that the conserved sites G_11_S_12_, G_133_K_134_, and D_138_K_139_R_140_ of SmpB were important for the interaction with SN.

### Selection of peptide aptamer PA-2 interacting with ArfA

**Figure 3:**
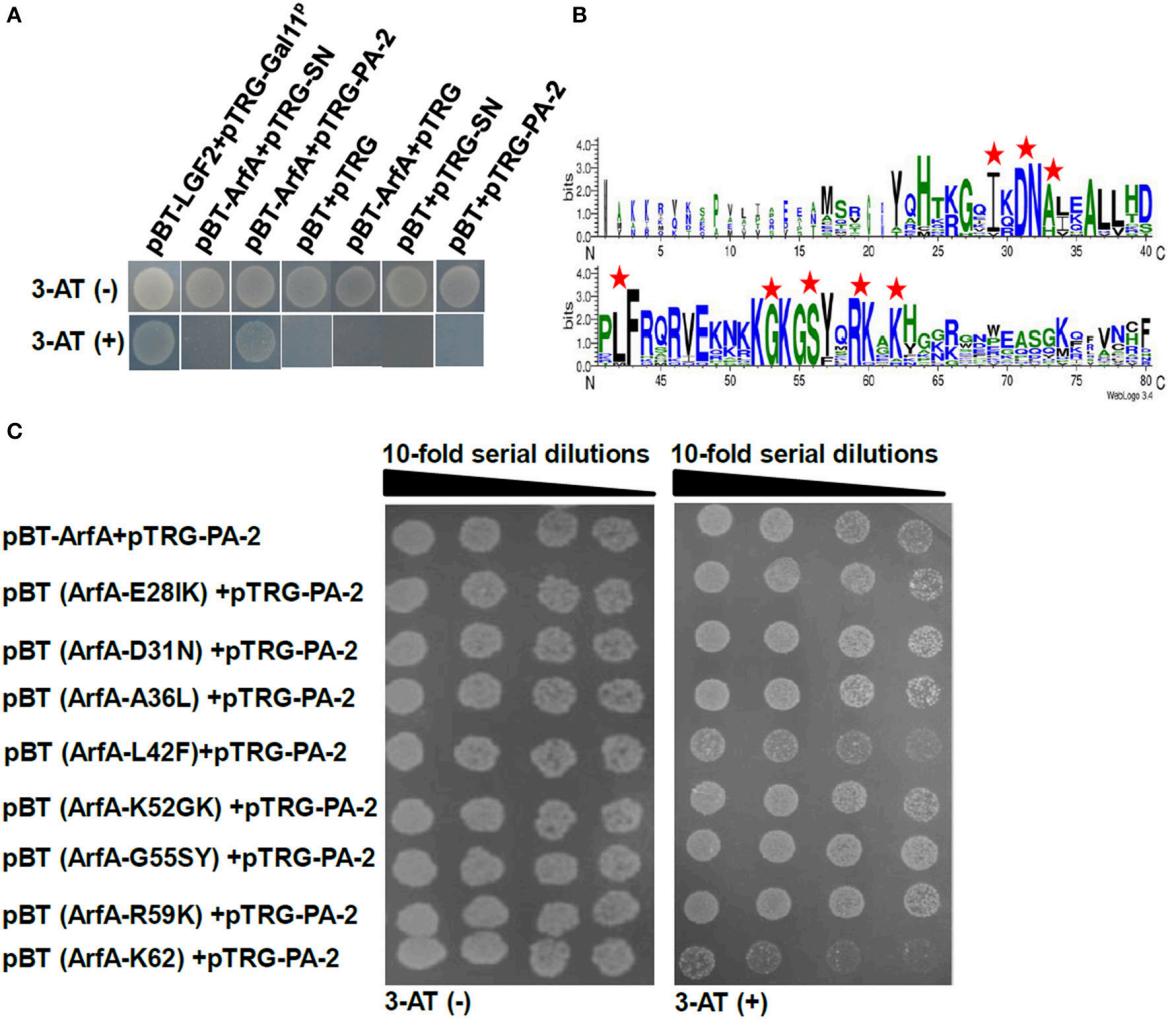
**Identification of key sites of ArfA interacting with PA-2**. The co-transformants were cultivated overnight, and spotted onto non-selective and 5 mM 3-AT selective medium by 10-series dilution method with 10 μl of 10^6^ CFU/ml as initiation. **(A)** Verification of surface loop of PA-2 interacting with ArfA. **(B)** Alignments of N- and C- terminuses of 15 ArfA sequences (Supplementary Table 4) were displayed by WebLogo (http://weblogo.berkeley.edu/logo.cgi). The mutation sites are marked by red pentagrams. **(C)** Verification of key sites of ArfA interacting with PA-2.

The peptide aptamer library was constructed to insert a random and exposed surface loop on the scaffold protein SN (Figure 1A), and seven specific peptide aptamers were selected and sequenced by employing ArfA as bait (Table 3). Since the co-transformants of pBT-ArfA and pTRG-PA-2 grew on 5 mM 3-AT medium, while the co-transformants conferring pBT-ArfA and pTRG-SN did not, showing that ArfA interacted with the surface loop particularly (Figure 3A). To further analyze the key sites of ArfA interacting with PA-2, the conservative amino acids of ArfA in *A. veronii* C4 were aligned with other pathogenic bacteria (Supplementary Table 4) using WebLogo (http://weblogo.berkeley.edu/logo.cgi) (Figure 3B). A series of pBT-ArfA mutants (Table 1) were co-transformed with pTRG-PA-2 for analyzing the key sites of interaction (Figure 3C). The results revealed the co-transformants of pBT (ArfA-K_62_) and pTRG-PA-2 had growth defect on 5 mM 3-AT medium, demonstrating that the residue K_62_ of ArfA was vital for interacting with PA-2.

**Table 3:** **The amino acid sequences of peptide aptamers binding with protein ArfA**.

**Peptide aptamers**	**Sequence**
PA-2	IGQEWGLGVRGPLSAK
PA-3	MGQVNSIQPAELRLVV
PA-5	PRDGIVSGSRLRGLHY
PA-6	STVFGVIEITRTLNST
PA-7	WTVRSAQAVEWSSVR
PA-10	MSTPWGSILARHLDTR
PA-12	VRCWVNTFPNGVHSWG

### Protein modeling and docking

**Figure 4:**
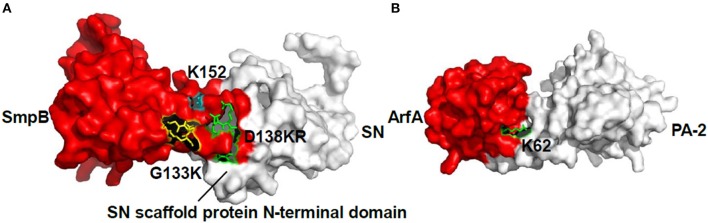
**The docking results of SmpB-SN complex and ArfA-PA-2 complex**. **(A)** Model of SmpB-SN complex was generated using HADDOCK server. SmpB was presented as a surface model with red color, and SN was presented as a surface model with white color. The residues G_133_K_134_, D_138_K_139_R_140_ and K_152_ were shown as stick models with yellow, green and blue colors respectively. **(B)** Model of ArfA-PA-2 complex was generated using HADDOCK server. ArfA was presented as a surface model with red color, and PA-2 was presented as a surface model with white color. The residue K_62_ was shown as a stick model with green color.

The HADDOCK between SmpB and SN grouped 163 structures into 13 clusters, which represented 81.5% of the water-refined models and the top cluster is the most reliable. Its Z-score indicated the standard deviations from the averages in the clusters. Thereinto, the best score is −1.9, and the docking results showed that the residues D_138_K_139_R_140_ and G_133_K_134_ interacted with the N-terminal region of SN (Figure 4A). The HADDOCK between ArfA and PA-2 grouped 149 structures into 14 clusters, which represented 74.5% of the water-refined models, and the best Z-score is −1.8, and also the docking result showed that the residue K_62_ interacted with PA-2 (Figure 4B).

### Growths of engineered *A. veronii* C4 strains at different temperatures

**Figure 5:**
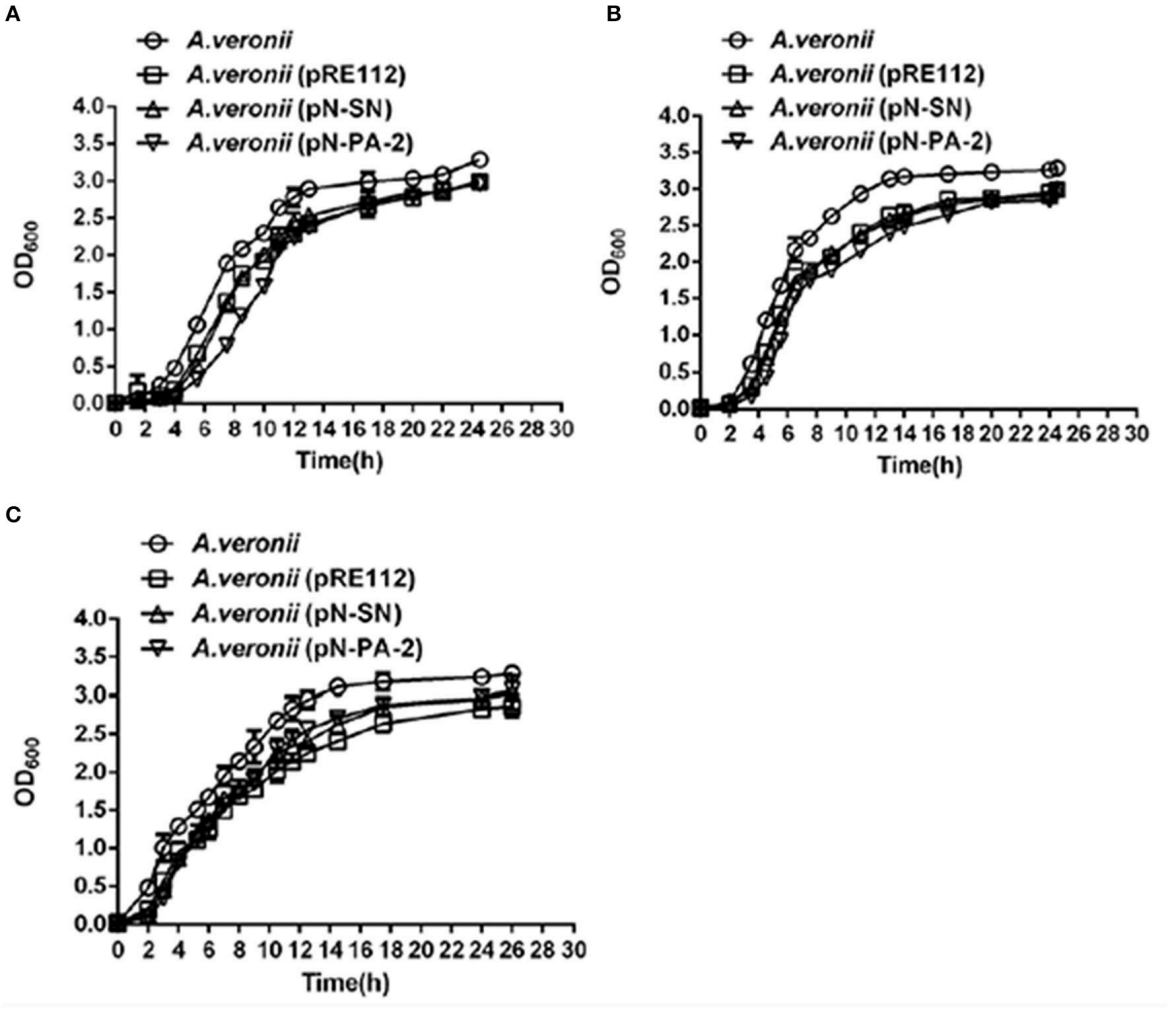
**Growth curves of ***A. veronii*** C4 derivatives at different temperatures**. *A. veronii* C4 derivatives were grown overnight and diluted to culture at an initial OD_600_ of 0.02 in LB supplemented with 50 μg/ml ampicillin, and samples were taken to measure at time intervals. Results were represented as mean values of three independent experiments with standard deviation (SD). **(A)** at 25°C; **(B)** at 30°C; **(C)** at 42°C.

To analyze whether SN and PA-2 affected the functions by interacting with SmpB and ArfA in *A. veronii* C4, pN-SN or pN-PA-2 were introduced into *A. veronii* C4 for expression, correspondingly. At 25°C, wild type *A. veronii* C4 had fastest growth, while *A. veronii* C4 (pN-SN) had slower growth as well as *A. veronii* C4 (pRE112), accompanied by severely impaired growth in *A. veronii* C4 (pN-PA-2) (Figure 5A). At 30°C, all engineered strains had consistent growth characteristics which grew slower than wild-type (Figure 5B), and the similar growth tendencies were observed in the engineered *A. veronii* C4 strains compared to wild type at 42°C (Figure 5C). Taken together, the results revealed that *A. veronii* C4 (pN-SN) and *A. veronii* C4 (pN-PA-2) grew defectively at different temperatures, signifying that expressions of SN and PA-2 could recognize and inhibit SmpB and ArfA *in vivo* individually.

### Growths of engineered *A. veronii* C4 strains at different concentrations of sugars or KCL

**Figure 6:**
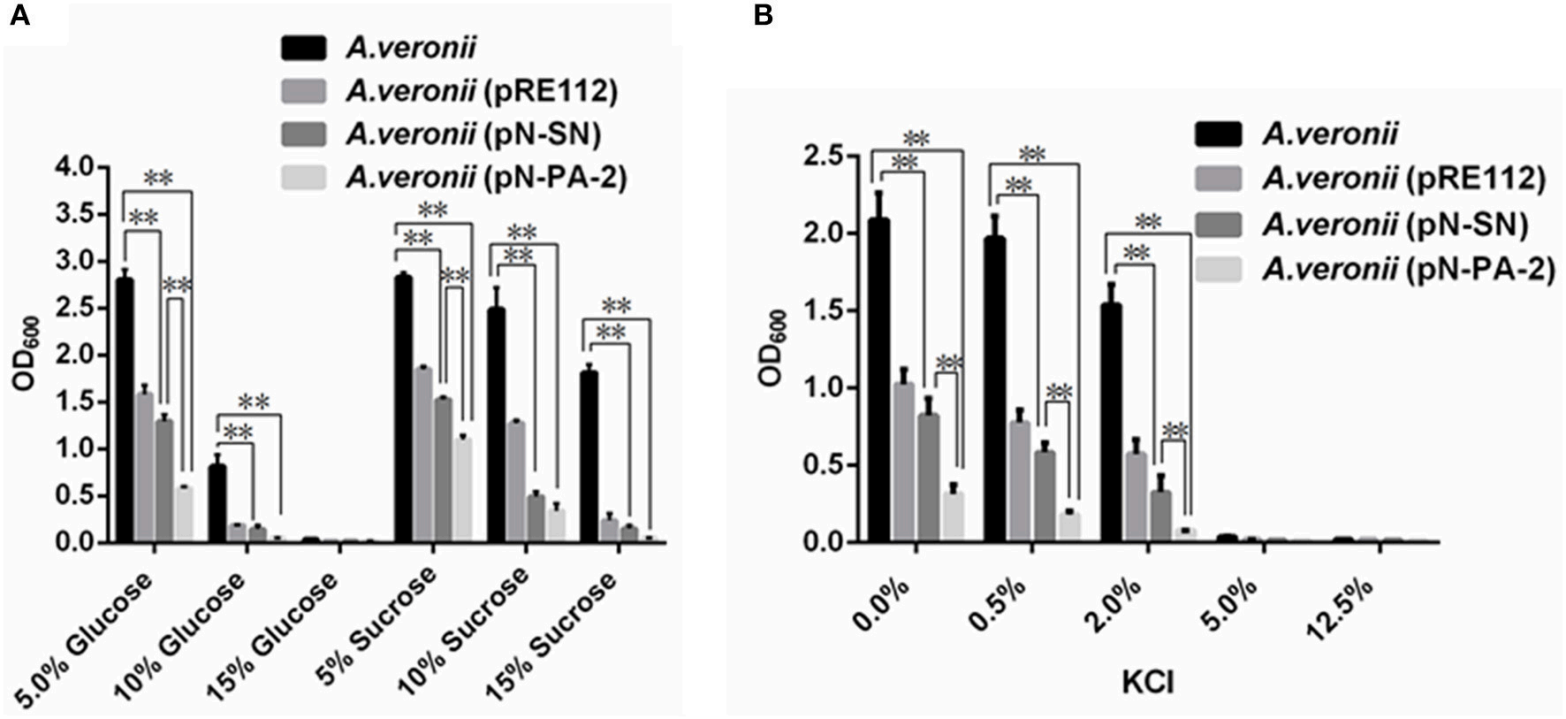
**Growths of engineered ***A. veronii*** C4 strains at different concentrations of sugars or KCl**. *A. veronii* C4 derivatives were grown overnight and diluted to an initial OD_600_ of 0.02 in LB supplemented with 50μg/ml ampicillin, and inoculated at different concentrations of glucose and sucrose and KCl for OD_600_ measurements at 12 h. Results were represented as mean values of three independent experiments with SD. The double asterisk was marked as extremely significant difference compared with wild type (*p* < 0.01). **(A)** Growths of *A. veronii* C4 derivatives at different concentrations of glucose and sucrose. **(B)** Growths of *A. veronii* C4 derivatives at different concentrations of KCl.

The growth levels of *A. veronii* C4 derivatives were evaluated under different concentrations of glucose and sucrose. The growths of *A. veronii* C4 (pN-SN) and *A. veronii* C4 (pN-PA-2) showed extremely significant differences compared with those of wild type at 5, 10, 15% glucose and sucrose (Figure 6A). Furthermore, the same concentration of glucose manifested slower than sucrose in all *A. veronii* C4 derivatives (Figure 6A). At 15% glucose, all of *A. veronii* C4 derivatives were completely suppressed. We specially analyzed the growth differences between *A. veronii* C4 (pN-SN) and *A. veronii* C4 (pN-PA-2) at 5% glucose and sucrose, demonstrating that they had extremely significant differences (*P* < 0.01) (Figure 6A). The growths of *A. veronii* C4 (pN-SN) and *A. veronii* C4 (pN-PA-2) displayed extremely significant differences compared with those of wild type at 0, 0.5, 2% KCl, respectively (*P* < 0.01) (Figure 6B). Besides, the growth of *A. veronii* C4 (pN-PA-2) appeared slower than that of *A. veronii* C4 (pN-SN) (*P* < 0.01) (Figure 6B). At 5 and 12.5% KCl, all of *A. veronii* C4 derivatives were completely suppressed. Taken together, the results indicated that knockdowns of SmpB and ArfA were deficient in the growths under glucose and sucrose stresses.

### Growths of engineered *A. veronii* at different antibiotics concentrations

**Figure 7:**
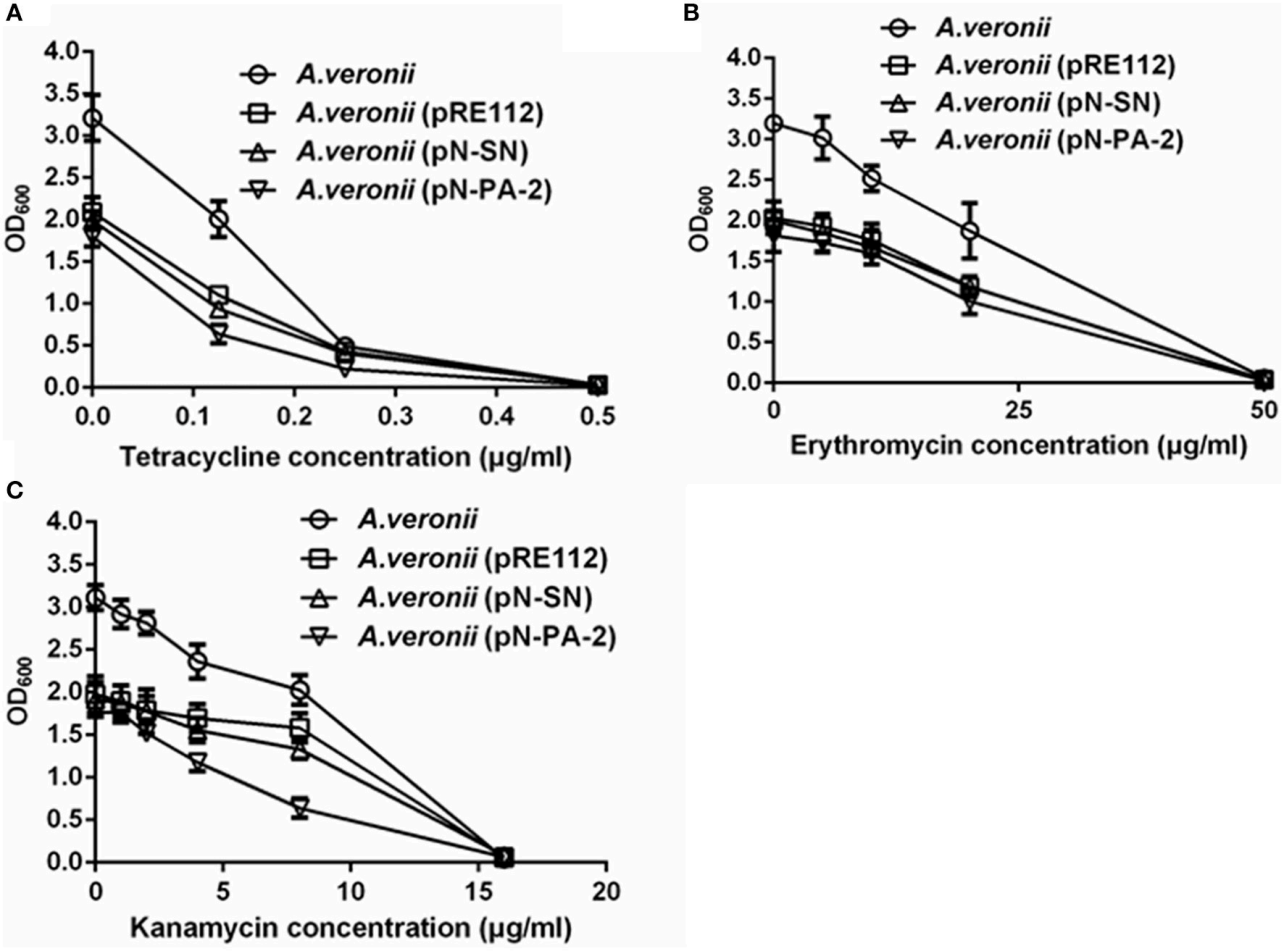
**Growths of engineered ***A. veronii*** C4 strains in different concentrations of antibiotics**. **(A)** Growth measurements of engineered *A. veronii* C4 strains at 12 h culture in 0–0.5 μg/ml tetracycline. **(B)** Growth measurements of engineered *A. veronii* C4 strains at 12 h culture in 0–50 μg/ml erythromycin. **(C)** Growth measurements of engineered *A. veronii* C4 strains at 12 h culture in 0–20 μg/ml kanamycin.

To assess the effects of the SmpB and ArfA on the growths under various antibiotics, the engineered *A. veronii* C4 strains were treated at tetracycline, kanamycin and erythromycin for measurements. The results showed that all engineered *A. veronii* C4 strains grew slower compared with wild type under various antibiotics (Figures 7A–C). Of particular note was that *A. veronii* C4 (pN-PA-2) retarded the growth severely compared to wild type, while *A. veronii* C4 (pRE112) and *A. veronii* C4 (pN-SN) impaired moderately at 0−15 μg/ml kanamycin (Figure 7C).

### Down-regulations of *rpoS* and *nhaP* transcriptions by introducing SN and PA-2 to *A. veronii* C4

**Figure 8:**
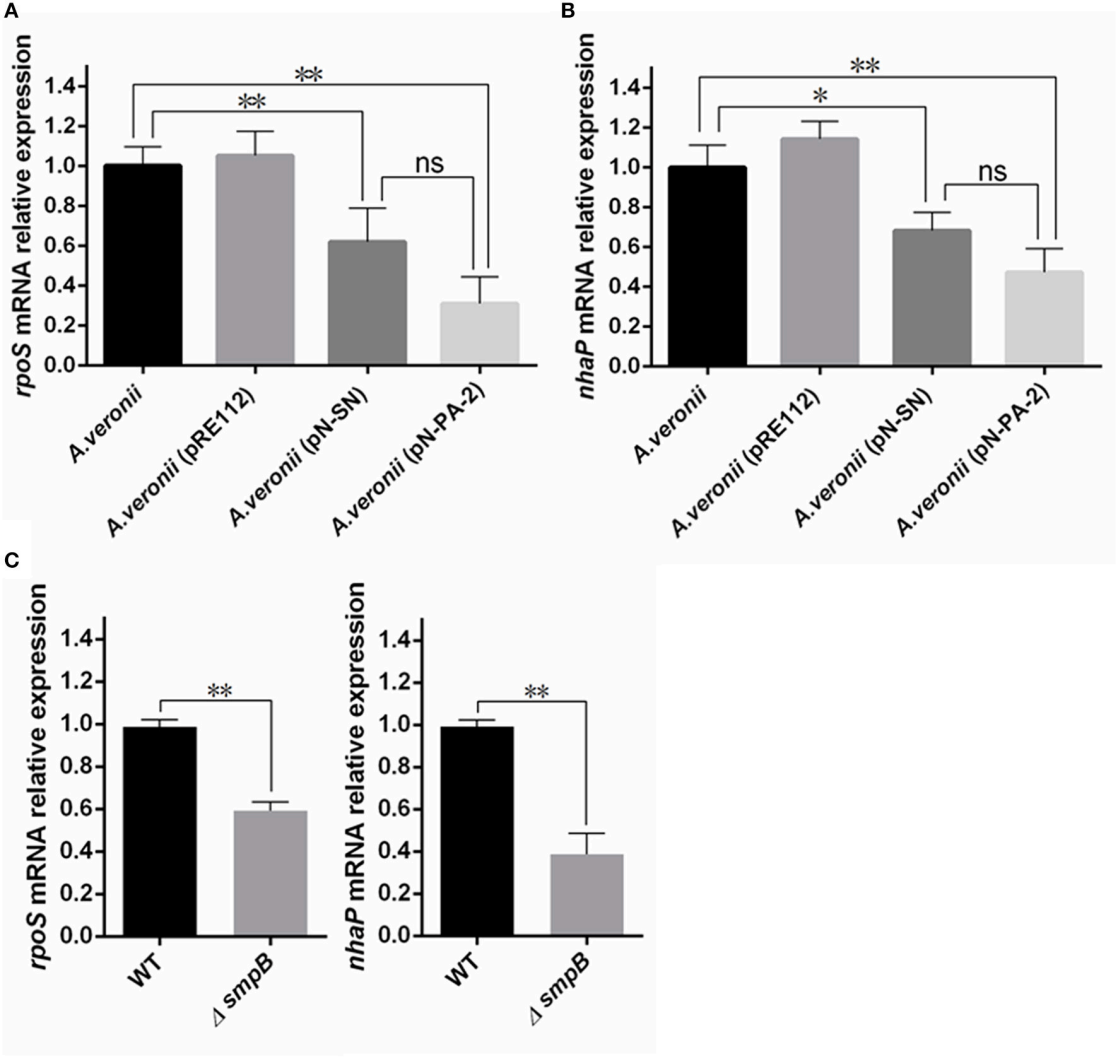
**Analysis of quantitative real time PCR (qRT-PCR)**. All *A. veronii* C4 derivatives were cultured to stationary phases at 30°C in LB containing 2% KCl and harvested for transcriptional quantification. Relative expression was normalized to 16S rRNA. The results were represented as mean values of three independent experiments with SD. The single and double asterisk represented significant (*p* < 0.05) and extremely significant difference (*p* < 0.01) compared with wild type, separately. **(A)** Relative expression analysis of *rpoS* gene. **(B)** Relative expression analysis of *nhaP* gene. **(C)** Relative expression analysis of *rpoS* and *nhaP* genes between wild type and *smpB* knockout.

As the growths of A. veronii C4 (pN-SN) and A. veronii C4 (pN-PA-2) had significant slower in comparison with wild type and A. veronii C4 (pRE112) under 2.0% KCl culture (*P* < 0.01) (Figure 6B), the total RNA were extracted for transcription analysis of *rpoS* and *nhaP* Figures 8A,B. The results implied that SN and PA-2 expressions might recognize and disturb the functions of SmpB and ArfA, leading to down-regulation of mRNA transcriptions of *rpoS* and *nhaP*, and eventually render the growth lags of *A. veronii* C4 (pN-SN) and *A. veronii* C4 (pN-PA-2) at 2.0% KCl in contrast to those of wild type and *A. veronii* C4 (pRE112). Similarly, the transcriptions of *rpoS* and *nhaP* in *smpB* knock-out strain were significantly down-regulated compared to wild type (*P* < 0.01) Figure 8C.

## Discussion

In bacteria, protein quality control systems play important roles in the cell survival, stress tolerance and virulence under adverse environments (Merdanovic et al., 2011). During the translation, mRNA may be truncated due to the mechanical defect, nuclease influence and mRNA processing (Keiler and Feaga, 2014). As a consequence, the bacterial ribosomes often stall at the end of aberrant mRNA lacking normal stop codons (Himeno et al., 2015). Since ribosome numbers are limited, the procrastinations give rise to deficits in the active numbers (Personne and Parish, 2014), and follow with the arrests of protein synthesis and products of toxic nascent peptides, and eventually affect the cell viability even to death. Therefore, bacteria evolve unique translation quality control systems to rescue the stalled ribosomes, by releasing the incomplete ribosomes from aberrant mRNA, degrading the nascent peptides with proteases, and deteriorating the damaged mRNAs. Among the stalled-ribosome rescue systems, there have alternative recued factors ArfA and YaeJ besides trans-translation pathway (Alumasa and Keiler, 2015), which is very important to ensure translation quality (Keiler and Shapiro, 2003). ArfA is first identified to compensate for the viability of *E. coli* in the absence of trans-translation (Chadani et al., 2010). Although ArfA is not essential, double mutations of both trans-translation and ArfA are synthetically lethal for *E. coli* (Garza-Sánchez et al., 2011).

Hence, we tempt to develop anti-microbial drug which target to the ribosome rescued factors for disturbing their functions and prevent the bacterial strains as vaccines. In this study, SN is chosen as scaffold protein to create the peptide aptamer library, since it has small size, folds automatically without chaperones, exposes a loop on its surface, and expresses as a soluble protein in prokaryotes (Norman et al., 1999). Peptide aptamers are combinatorial molecules with the insertion of the individually alterable peptide into the scaffold protein, resulting in a constrained surface loop which binds to their targets with strong affinity and high specificity (Hoppe-Seyler and Butz, 2000). Compared with antibodies, peptide aptamers are smaller in size while possessing similar binding property to targets both *in vitro* and *in vivo* (Skerra, 2007).

SmpB or ArfA were inserted into pBT vector as baits separately, and used to screen from peptide aptamer library. Eventually the peptide aptamer PA-2 was selected where the surface loop interacted with ArfA (Figure 3A), and SN scaffold protein interacted with the C-terminus of SmpB (Figure 2C). Previous studies reveals that unstructured C-terminal tail of SmpB acts imperatively in trans-translation process (Kurita et al., 2010; Miller et al., 2011), suggesting that PA-2 might interact and demonstrate to inhibit both SmpB and ArfA effectively.

However, our primary results showed that the purified SN and PA-2 never exhibited stable bacteriostatic activities *in vitro* when treating in *A. veronii* C4 (Data not shown), because their sizes could not allow to penetrate the bacteria cell wall for interacting with SmpB and ArfA, which acted as cytoplasmic proteins *in vivo* (Russell and Keiler, 2009). Besides, our results showed that plasmids pRE112 and pk18mobsacB could replicate autonomously in *A. veronii* C4 (Data not shown), prompting us to construct the vectors pN-SN or pN-PA-2 for expression *in vivo*, in which utilizing neokanamycin promoter of pk18mobsacB for exogenous expression and replication origin of pRE112 for amplification (Sichwart et al., 2011). After the vectors pRE112 or pN-SN or pN-PA-2 was transformed into *A. veronii* C4 individually, the growths were compared and analyzed under stresses.

Especially at low temperature, the strains cause extended lag to recognize translation intermediates, thereby resulting in reduced rates of translation (Somogyi et al., 1993), and ribosome stalling after defective translocations (Himeno et al., 2015). On this count, SmpB and ArfA contributed most at 25°C, and *A. veronii* C4 (pN-PA-2) showed more observable retardation of growth in all engineered *A. veronii* C4 strains (Figure 5A). In normal culture condition, SmpB and ArfA were abundant in rescuing the translation malfunctions (Keiler, 2008), so no matter whether SmpB and ArfA were interfered using SN and PA-2 at 30°C, the growth tendencies of *A. veronii* C4 (pN-SN) and *A. veronii* C4 (pN-PA-2) were accordant by contrast with *A. veronii* C4 (pRE112) (Figure 5B). At 42°C, all the engineered *A. veronii* C4 strains had similar growth curves, but possessed growth defects compared to wild type (Figure 5C). It probably ascribes to enhanced expressions of heat shock proteins, maintaining the growths at high temperatures (Yamamori and Yura, 1982). Current study reports that heat-shock protein GTPase HflX conducts as an alternative ribosome-recycling factor which involving in stalled-ribosome rescue during heat-shock, and compensates for the disturbances of trans-translation and ArfA (Zhang et al., 2015).

When *A. veronii* C4 and its derivatives thereof were treated by parallel concentrations of sucrose or glucose, the strains always grew better in sucrose than those in glucoses (Figure 6A). One possible explanation for these findings is that, sucrose is a kind of non-reducing disaccharides, which protecting the bacterial membrane integrity and avoiding the death, while glucose cannot because of its characteristics as reducing monosaccharide (Giulio et al., 2005).

To explain why the engineered *A. veronii* C4 had weak growths under adverse conditions containing antibiotics, sugars, and KCl (Figures 6, 7), the transcription levels of *rpoS* and *nhaP* were quantified by RT-PCR (Figure 8). RpoS protein is the σ subunit of RNA polymerase in regulation of group-specific genes expressions during stress response and stationary phase (Hengge-Aronis, 1993, 1996; Battesti et al., 2011), and bacteria defective in *rpoS* gene for RpoS synthesis have proved to be more sensitive to different adverse conditions (Chen and Jiang, 2014). For example, RopS has a vital role in cell survival under a high salt stress in *E. coli* (Tarusawa et al., 2016), because trans-translation regulates RpoS expression by preventing the ribosome stalling (Hong et al., 2005; Ranquet and Gottesman, 2007). The mRNA level of *rpoS* gene was down-regulated (Figure 8A) in *A. veronii* C4 (pN-SN) and *A. veronii* C4 (pN-PA-2), inferring the expressions of SN and PA-2 could interact and knockdown the functions of SmpB and ArfA, which resulted to growth defects under KCl stress. The gene *nhaP* encodes NhaP-type Na^+^/H^+^ or K^+^/H^+^ antiporter, which avoids excessive accumulation of potassium in the cytoplasm (Resch et al., 2010). Similarly, the expressions of SN and PA-2 might interrupt the functions of SmpB and ArfA, following with the down-regulation of *nhaP* mRNA in *A. veronii* C4 (pN-SN) and *A. veronii* C4 (pN-PA-2) (Figure 8B). To further confirm that *smpB* gene regulated *rpoS* and *nhaP* directly, the transcription levels of *rpoS* and *nhaP* were compared between wild type and *smpB* knock-out strain, supplying that the deletion mutant of SmpB was accompanied by low expressions of RopS and NhaP (Figure 8C).

In summary, peptide aptamer PA-2, in which scaffold protein SN interacted with SmpB and the surface loop interacted with the ArfA, was screened from the peptide aptamer library. It binds primarily to G_11_S_12_, G_133_K_134_, and D_138_K_139_R_140_ of SmpB and K_62_ of ArfA for disturbing SmpB and ArfA actions, bringing about the interferes with their partner or substrates in *A. veronii* C4.When expressed SN and PA-2 in *A. veronii* C4, *A. veronii* C4 (pN-SN) and *A. veronii* C4 (pN-PA-2) exerted distinct growth defects at 25°C. Analogously, the growths of *A. veronii* C4 (pN-SN) and *A. veronii* C4 (pN-PA-2) showed significantly lower compared to those of wild type and *A. veronii* C4 (pRE112) by treating with glucose, sucrose, kanamycin and KCl. Further on, the down-regulated transcriptions of *rpoS* and *nhaP* genes eventually set off the growth defects, when employing PA-2 to disturb the functions of SmpB and ArfA. Collectively, the engineered *A. veronii* C4 (pN-PA-2) could be potentially attenuated vaccine for the prevention of *A. veronii* pathogen, and the peptide aptamer PA-2 could theoretically develop as anti-microbial drugs targeted to the ribosome rescued factors.

## Author contributions

YZ and ZL designed the experiments. PL, YC, DW, YT, HS, and HT performed and analyzed for the work. PL, QS, YZ, and ZL interpreted the data. ZL, PL, and DW drafted the work. All the authors agreed to submit for Frontiers in Microbiology Journal.

### Conflict of interest statement

The authors declare that the research was conducted in the absence of any commercial or financial relationships that could be construed as a potential conflict of interest.
