# Supplementary material for: Genetic Selection of Peptide Aptamers That Interact and Inhibit Both Small Protein B and Alternative Ribosome-Rescue Factor A of Aeromonas veronii C4
Source: Front Microbiol. 2016 Aug 18;7:1228. doi: 10.3389/fmicb.2016.01228 (PMC4988972; doi:10.3389/fmicb.2016.01228)
Supplement: Supplementary file 1 [file Image1.PDF]

MGYPYDVPDYASLPGIQPATSTKKLHKEPATLIKAIDGTTVKLMYKGQPMTFRLLLVDTPE
FGGVTFLVNTYPNGVQSRAGGRSKYGPEASAFTKKMVENAKKIEVELDKGQRTDKYGR
GLAYIYADGKMVNEALVRQGLAKVAYVYKPKNTHEQHLRKSEAQAKKEKLNIWSEDNA
DSGQVDID

Supplement Figure 1丨The amino acid sequence of scaffold protein SN.
