# Supplementary material for: Genetic Selection of Peptide Aptamers That Interact and Inhibit Both Small Protein B and Alternative Ribosome-Rescue Factor A of Aeromonas veronii C4
Source: Front Microbiol. 2016 Aug 18;7:1228. doi: 10.3389/fmicb.2016.01228 (PMC4988972; doi:10.3389/fmicb.2016.01228)
Supplement: Supplementary file 2 [file Image2.PDF]

MSKKNSKNKAGSSTIALNRTARHEYFIEEKIEAGLSLQGWEVKSLRAGKANISEAYVIFRD
GEAYLFGSSFLPLQAASSHVVCDPTRTRKLLLSRRELDKLESLIARQGYTIVPLALYWKQC
WVKVEIGLVKGKKEHDKREDTKAREWDREKARIMKNKHRG

**Supplement Figure 2丨The amino acid sequence of SmpB protein**.
