# Supplementary material for: Genetic Selection of Peptide Aptamers That Interact and Inhibit Both Small Protein B and Alternative Ribosome-Rescue Factor A of Aeromonas veronii C4
Source: Front Microbiol. 2016 Aug 18;7:1228. doi: 10.3389/fmicb.2016.01228 (PMC4988972; doi:10.3389/fmicb.2016.01228)
Supplement: Supplementary file 4 [file Image4.PDF]

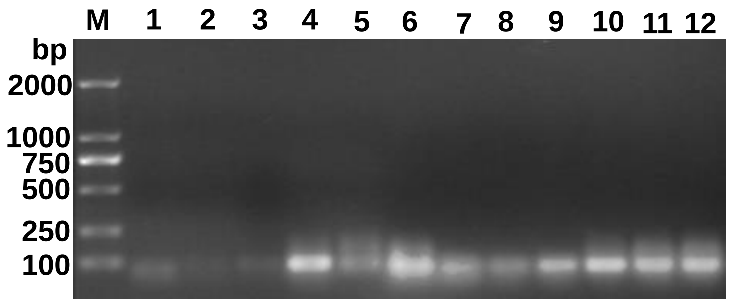

Supplement Figure 4 | Optimized PCR reaction for high yield and quality of double-stranded oligonucleotides. M: DNA molecular weight marker DL2000; 1: Negative control; 2: 43 ℃, 5 cycles; 3: 43 ℃, 8 cycles; 4: 43 ℃, 12 cycles; 5: 43 ℃, 16 cycles; 6: 43 ℃, 20 cycles; 7: 46 ℃, 0 cycles; 8: 46 ℃, 5 cycles; 9: 46 ℃, 8 cycles; 10: 46 ℃, 12 cycles; 11: 46 ℃, 16 cycles; 12: 46 ℃, 20 cycles.

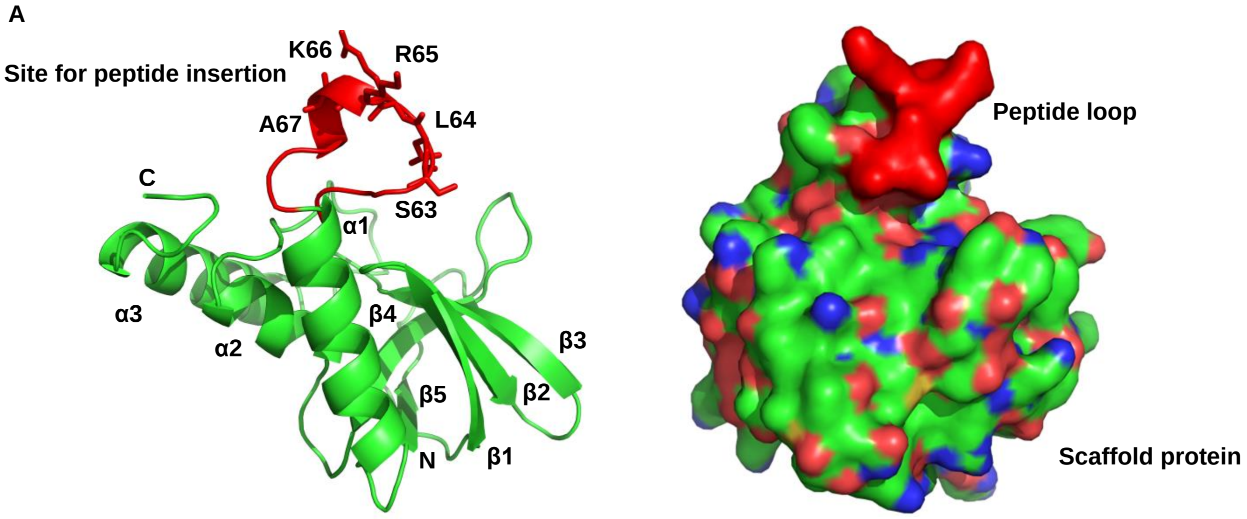
A
Site for peptide insertion
K66
R65
A67
L64
S63
C
α1
α3
α2
β4
β3
β5
β2
N
β1
Peptide loop
Scaffold protein
