# Supplementary material for: Genetic Selection of Peptide Aptamers That Interact and Inhibit Both Small Protein B and Alternative Ribosome-Rescue Factor A of Aeromonas veronii C4
Source: Front Microbiol. 2016 Aug 18;7:1228. doi: 10.3389/fmicb.2016.01228 (PMC4988972; doi:10.3389/fmicb.2016.01228)
Supplement: Supplementary file 5 [file Table1.DOCX]

**Supplement Table 1 | Primers in this study**

| **Primers** | **Sequnce(5’-3’)** | **Enzyme** |  |
| --- | --- | --- | --- |
| pBT-ArfA | F1:5’-CG*GAATTC*CATGGCCAATATTCGGGTCAA-3’  R1:5’-GA*AGATCT*TCAGCAGCAGACGCGGATC-3’ | *Eco*R I  *Bgl* II |  |
| pTRG-SN  pTRG-SN ΔC45  pTRG-SN ΔN57 | F2:5’-CCG*CTCGAG*GCGGCCGCAATGGGTTACCCATACGACGTTC-3’  R2:5’-GATCTC*ACTAGT*TTAGTGGTGGTGGTGGTGGTGGTCGATGTC-3’  F3:5’-CCG*CTCGAG*ATGGGTTACCCATACGACGT-3’  R3:5’-G*ACTAGT*TTACAAACCTTGTCTAACCAAAGC-3’  F4:5’-CCG*CTCGAG*GACACCCCAGAATTCTCCTTA-3’  R4:5’-G*ACTAGT*TTAGTCGATGTCAACTTGACCAG-3’  F5:5’-CC*CCCGGG*AGCGCAAAGAGAAAGCAGGTAG-3’  R5:5’-GC*TCTAGA*TCAGAAGAACTCGTCAAGAAGG-3’ | *Xho* I  *Spe* I  *Xho* I  *Spe* I  *Xho* I  *Spe* I  *Xma* I  *Xba* I |  |
| pN-SN | F6:5’-CC*CCCGGG*AGCGCAAAGAGAAAGCAGGTAG-3’  R6:5’-AACGTCGTATGGGTAACCATCTTGTTCAATCATGCG-3’  F7:5’-CGCATGATTGAACAAGATGGTTACCCATACGACGTT-3’  R7:5’-GC*TCTAGA*GGATCTCACTAGTTTAG-3’ | *Xma* I  *Xba* I |  |
| CamR | F8:5’-TAAATACCTGTGACGGAAG-3’  R8:5’-ACATCACTTATTCAGGCGTAG-3’ |  |  |
| Peptide Aptamer  pBT  pTRG | F9:5’-CCC*GAATTC*GGTGGT-3’  R9:5’-GTACTT*AGATCT*ACCAC-3’  F10:5’-TCCGTTGTGGGGAAAGTTATC-3’  R10:5’- GGGTAGCCAGCAGCATCC-3’  F11:5’-TGGCTGAACAACTGGAAGCT-3’  R11:5’-ATTCGTCGCCCGCCATAA-3’ | *Eco*R I  *Bgl* II |  |
| **Mutated Residue** | **Primers Sequence (5’-3’)** | | |
| pBT (ArfA-E28IK)  pBT (ArfA-D31N)  pBT (ArfA-A36L)  pBT (ArfA-L42F)  pBT (ArfA-K52GK)  pBT (ArfA -G55SY)  pBT (ArfA -R59K)  pBT (ArfA-K62) | F12:5’-CAACTATGCCCATCAGCGCGCAGCAGCAAAGGATAACCATCTGCATGCC-3’  R12:5’-GGCATGCAGATGGTTATCCTTTGCTGCTGCGCGCTGATGGGCATAGTTG-3’  F13:5’-TCAGCGCGGCGAGATAAAGGCAGCACATCTGCATGCCCTGCTCT-3’  R13:5’-AGAGCAGGGCATGCAGATGTGCTGCCTTTATCTCGCCGCGCTGA-3’  F14:5’-GATAAAGGATAACCATCTGCATACCGCAGCATCTGATCCGCTGTTTCG-3’  R14:5’-CGAAACAGCGGATCAGAGAGTGCTGCATGCAGATGGTTATCCTTTATC-3’  F15:5’-GCCCTGCTCTCTGATCCGGCAGCACGCAGCCGGGTCGAGCG-3’  R15:5’- CGCTCGACCCGGCTGCGTGCTGCCGGATCAGAGAGCAGGGC-3’  F16:5’-CGGGTCGAGCGCAACAAGGCAGCAGCAAAGGGGAGTTACCAGCGCA-3’  R16:5’-TGCGCTGGTAACTCCCCTTTGCTGCTGCCTTGTTGCGCTCGACCCG-3’  F17:5’-GCGCAACAAGAAGGGCAAGGCAGCAGCACAGCGCAAGGCCAAATTCG-3’  R17:5’-CGAATTTGGCCTTGCGCTGTGCTGCTGCCTTGCCCTTCTTGTTGCGC-3’  F18:5’-GGCAAGGGGAGTTACCAGCGCAGCAGCCAAATTCGGCAAGCGGT-3’  R18:5’-ACCGCTTGCCGAATTTGGCTGCTGCGCTGGTAACTCCCCTTGCC-3’  F19:5’-AGTTACCAGCGCAAGGCCAGCATTCGGCAAGCGGTGGGAG-3’  R19:5’-CTCCCACCGCTTGCCGAATGCTGGCCTTGCGCTGGTAACT-3’ | | |

The underlined Nucleotide indicates position of mutation
