# Supplementary material for: Genetic Selection of Peptide Aptamers That Interact and Inhibit Both Small Protein B and Alternative Ribosome-Rescue Factor A of Aeromonas veronii C4
Source: Front Microbiol. 2016 Aug 18;7:1228. doi: 10.3389/fmicb.2016.01228 (PMC4988972; doi:10.3389/fmicb.2016.01228)
Supplement: Supplementary file 6 [file Table2.DOCX]

**Supplement Table 2 | Primers for real time PCR**

| **Gene** | **Primers for Real-time PCR** |  |
| --- | --- | --- |
| 16S rRNA | F20:5’-ATTCCAGGTGTAGCGGTGAA-3’  R20:5’-CACAGCCTCCAAATCGACAT-3’ |  |
| *rpoS* | F21:5’-GACGCTGAACCCGAATCT-3’  R21:5’-GTGGCATCCATCACCTTGT-3’ |  |
| *nhaP* | F22:5’-CCTTCCTCATCGGCTACG-3’  R22:5’-TACGGCAATCGGGTCAGT-3’ |  |
