# Supplementary material for: Genetic Selection of Peptide Aptamers That Interact and Inhibit Both Small Protein B and Alternative Ribosome-Rescue Factor A of Aeromonas veronii C4
Source: Front Microbiol. 2016 Aug 18;7:1228. doi: 10.3389/fmicb.2016.01228 (PMC4988972; doi:10.3389/fmicb.2016.01228)
Supplement: Supplementary file 7 [file Table3.DOCX]

### Supplement Table 3. NCBI accession numbers of SmpB protein in pathogenic bacteria

| Bacterial Strain | NCBI accession no. |
| --- | --- |
| *Aeromonas salmonicida* NBRC 13784 | GAJ49632.1 |
| *Aeromonas hydrophila* SSU | EKB29326.1 |
| *Vibrio albensis* VL426 | EEO04069.1 |
| *Escherichia coli* K-12 | ACQ76411.1 |
| *Shigella boydii* Sb227 | ABB67282.1 |
| *Salmonella enterica* | KOP04525.1 |
| *Klebsiella pneumoniae* SB3432 | CCI75561.1 |
| *Raoultella ornithinolytica* 10-5246 | EHT06444.1 |
| *Enterobacteriaceae bacterium* LSJC7 | WP_026059282 |
| *Yersinia pestis* KIM D27 | EFA46562.1 |
| *Pantoea ananatis* AJ13355 | BAK12282.1 |
| *Haemophilus influenzae* KR494 | AGV11438.1 |
| *Pasteurella bettyae* CCUG 2042 | EIJ69731.1 |
| *Neisseria gonorrhoeae* 1291 | EEH61951.1 |
| *Halomonas* sp. HL-93 | KPQ19636 |
