# Supplementary material for: Genetic Selection of Peptide Aptamers That Interact and Inhibit Both Small Protein B and Alternative Ribosome-Rescue Factor A of Aeromonas veronii C4
Source: Front Microbiol. 2016 Aug 18;7:1228. doi: 10.3389/fmicb.2016.01228 (PMC4988972; doi:10.3389/fmicb.2016.01228)
Supplement: Supplementary file 8 [file Table4.DOCX]

### Supplement Table 4. NCBI accession numbers of ArfA protein in pathogenic bacteria

| Bacterial Strain | NCBI accession no. |
| --- | --- |
| *Aeromonas salmonicida* NBRC 13784 | GAJ49277.1 |
| *Aeromonas hydrophila* SSU | EKB25641.1 |
| *Vibrio albensis* VL426 | EEO01365.1 |
| *Escherichia coli* K-12 | AKK14913.1 |
| *Shigella boydii* Sb227 | ABB67773.1 |
| *Salmonella enterica* | GAR68490.1 |
| *Klebsiella pneumoniae* SB3432 | CCI79101.1 |
| *Raoultella ornithinolytica* 10-5246 | EHT04880.1 |
| *Enterobacteriaceae bacterium* LSJC7 | WP_017374134.1 |
| *Yersinia pestis* KIM D27 | EFA47540.1 |
| *Pantoea ananatis* AJ13355 | BAK12898.1 |
| *Haemophilus influenzae* KR494 | AGV10897.1 |
| *Pasteurella bettyae* CCUG 2042 | EIJ67258.1 |
| *Neisseria gonorrhoeae* 1291 | EEH62293.1 |
| *Halomonas* sp. HL-93 | KPQ19636 |
